# Genomic Evaluation of *Coffea arabica* and Its Wild Relative *Coffea racemosa* in Mozambique: Settling Resilience Keys for the Coffee Crop in the Context of Climate Change

**DOI:** 10.3390/plants12102044

**Published:** 2023-05-20

**Authors:** Inocência da Piedade Ernesto Tapaça, Lopes Mavuque, Riccardo Corti, Samuele Pedrazzani, Ivete S. A. Maquia, Castigo Tongai, Fábio Luiz Partelli, José C. Ramalho, Isabel Marques, Ana I. Ribeiro-Barros

**Affiliations:** 1 Forest Research Center (CEF), Associate Laboratory TERRA, Instituto Superior de Agronomia (ISA), Universidade de Lisboa (UL), Tapada da Ajuda, 1349-017 Lisbon, Portugal; inoctapaca@gmail.com (I.d.P.E.T.); mavuque@gmail.com (L.M.); riccardo.corti@stud.unifi.it (R.C.); samuele.pedrazzani@stud.unifi.it (S.P.); ivetemaquia@gmail.com (I.S.A.M.); tongaicastigo@gmail.com (C.T.); cochichor@isa.ulisboa.pt (J.C.R.); 2 Mozambique Agricultural Research Institute (IIAM), Avenida das FPLM 2698, Mavalane B, Maputo P.O. Box 3658, Mozambique; 3 Unilurio, Faculty of Agricultural Sciences Campus de Unang, EN733 Km 42, Unango P.O. Box 3003, Mozambique; 4 Facoltà di Agraria, Università degli studi di Firenze, Piazzale delle Cascine 18, 50144 Firenze, Italy; 5 Biotechnology Center, Eduardo Mondlane University, Km 1.5, Maputo P.O. Box 3453, Mozambique; 6 Department of Scientific Services, Gorongosa National Park, Gorongosa P.O. Box 1983, Mozambique; 7 Centro Universitário do Norte do Espírito Santo (CEUNES), Departmento Ciências Agrárias e Biológicas (DCAB), Universidade Federal Espírito Santo (UFES), Rodovia BR 101 Norte, Km 60, Bairro Litorâneo, São Mateus 29932-540, ES, Brazil; partelli@yahoo.com.br; 8 Unidade de Geobiociências, Geoengenharias e Geotecnologias (GeoBioTec), Faculdade de Ciências e Tecnologia (FCT), Universidade NOVA de Lisboa (UNL), Monte de Caparica, 2829-516 Caparica, Portugal

**Keywords:** agroforestry systems, coffee, genetic diversity, microsatellites, SNPs, Mozambique

## Abstract

Climate change is negatively affecting the coffee value chain, with a direct effect on approximately 100 million people from 80 countries. This has been attributed to the high vulnerability of the two-mainstream species, *Coffea arabica* and *Coffea canephora*, to extreme weather events, with notable uneven increases in market prices. Taking into account the narrow genetic plasticity of the commercial coffee cultivars, wild-relatives and underutilized *Coffea* species are valuable genetic resources. In this work, we have assessed the occurrence of *Coffea* species in to understand the degree of genetic relationships between *Coffea* species in the country, as well as the patterns of genetic diversity, differentiation, and genetic structure. Only one wild species was found, *C. racemosa*, which showed a high level of genetic separation with *C. arabica*, based on plastid, as well as SSR and SNP analysis. *C. arabica* presented low levels of diversity likely related to their autogamous nature, while the allogamous *C. racemosa* presented higher levels of diversity and heterozygosity. The analysis of the functional pathways based on SNPs suggests that the stress signaling pathways are more robust in this species. This novel approach shows that it is vital to introduce more resilient species and increase genomic diversity in climate-smart practices.

## 1. Introduction

Coffee (*Coffea* L.) plays a prominent agricultural, social, and commercial role, standing as one of the world’s largest agricultural supply chains. The livelihoods of almost 25 million people are directly dependent on coffee cultivation, and more than 100 million people in over 80 countries are involved across the entire value chain [1]. The coffee market is also growing due to increasing consumption in emerging economies and a stronger interest in specialty coffee [1]. Despite the expansion of the coffee sector, the market relies mostly on genotypes from two species: *Coffea arabica* L. (Arabica coffee), which dominates the world market, and one of its ancestors, *Coffea canephora* Pierre ex A Froehner (Robusta coffee) [2,3]. However, the two-mainstream species are highly sensitive to changing temperatures and water availability [2,4,5], with visible impacts across the coffee value chain [1,6]. Traditionally, Arabica cultivars have an optimal annual mean temperature ranging between 18–21 °C, with temperatures above 23 °C accelerating fruit ripening, which can cause bean quality loss [7]. Seasonal high temperatures above 33 °C and dryer seasons can also reduce floral initiation and increase the production of abnormal reproductive structures and flower abortion [8]. Drought decreases coffee yield and quality, especially in Robusta coffee [9]. The effects of drought are also aggravated in Arabica plantations under full sunlight exposure [10]. According to the International Coffee Organization, 2020 and 2021 were already marked by a global reduction of Arabica and Robusta stocks associated with the vulnerability of these species to extreme weather events, with notable uneven increases in market prices [1]. The future seems not to be better as modeling analyses predict that the supply chain will be severely affected by climate change across this century, with significant effects on coffee yield and quality [10,11,12,13].

The quite low levels of genetic variation found in most commercial coffee cultivars [14] constitute a major concern regarding the long-term sustainability of the sector since there might not be enough genomic resilience to keep pace with climatic change [15]. Looking back at coffee, wild relatives offer the potential to increase the adaptive capacity of agricultural systems to diseases and climatic pressures, representing a large pool of new, untapped, genetic variation [16,17,18]. Therefore, considering the global environmental and anthropogenic scenario, as well as the narrow genetic plasticity of commercial coffee cultivars, several approaches are being implemented to ensure the sustainability of this important crop. The introduction of wild relatives and underutilized species, such as *Coffea racemosa* Lour., *Coffea zanguebariae* Lour., or *Coffea liberica* Hiern in the value chain, has been pointed out as one of the most promising approaches [19]. These species are considered highly resilient to environmental pressures, particularly high temperatures, and extreme precipitation events [19,20,21,22]. Additionally, *C. racemosa* (and probably *C. zanguebariae*, which is often misclassified as *C. racemosa*) shows relevant resistance to several pests and diseases [19,21,23]. Such tolerance to abiotic and biotic stresses, together with the short ripening period and unique flavor attributes, make these species an outstanding gene pool, as well as an important resource for new coffee blends [19]. 

Another recommended approach to mitigate climate change impacts on the coffee crop is the shift from intensive production under the full sun (and monocrop systems) to agroforestry systems (AFS) using native or other economic-important trees for shade, constituting refuges for biodiversity and providing multiple ecosystem services (e.g., timber, food, carbon sequestration, or nutrient cycling) [10,24,25,26]. The effectiveness of this strategy, which provides a better micro-environment for coffee plants, is however dependent on several factors, such as the agroecological conditions, shade density, crop management, and the cultivars used [27,28]. Successful coffee AFS systems have been implemented in several countries in the Americas and Africa countries [10,24,26,29,30]. Among them, the coffee AFS system established in the Gorongosa Mountain, which is part of the Gorongosa National Park (GNP) in Mozambique, has been pointed out as one of the most emblematic cases, not only in terms of the coffee crop sustainability but also regarding the positive socio-economic benefits to local communities, with a direct impact in the reversion of the accelerated loss and degradation of the tropical rainforest [10,26].

Located in southern Africa, Mozambique might contribute significantly to the coffee value chain, although it is currently not included in the list of coffee-producing countries: (i) it is a promising source of coffee wild-relatives [19,20,23], and (ii) it has a remarkable abundance of native forests [31]. A recent molecular study solved ambiguities between *C. racemosa* and *C. zanguebariae* and elucidated their distribution in northeastern Mozambique (Cabo Delgado Province) [19]. The same authors pinpointed the knowledge gap regarding the current distribution of these species in the rest of the country. Indeed, cultivation of *C. racemosa* in central Mozambique (Inhambane Province) was first reported in 1876 [32], and the last full description dates from 1973 [20]. More recently, a new study mapped the distribution and suitability of *C. arabica* plantations across the country [10]. Four regions (Manica, Sofala, Zambezia, and Nampula) were identified as suitable for Arabica, particularly under AFS management. 

**Figure 1:**
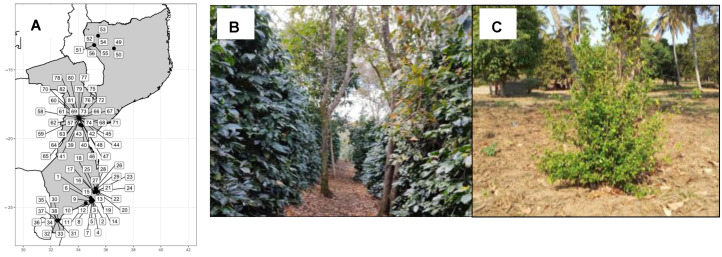
(**A**). Sampling of *Coffea* species in Mozambique. See Table S1 for the ID of samples. (**B**). Cultivation of *Coffea arabica* in the Gorongosa Mountain under agroforestry systems. (**C**). Wild plants of *Coffea racemosa*.

In this study, we have assessed, for the first time, the occurrence of *Coffea* species in southern and central Mozambique (Figure 1A) and the impact of genetic diversity on the long-term sustainability of the AFS implemented in Gorongosa Mountain. Specifically, we first aimed at understanding the degree of genetic relationships between *Coffea* species in Mozambique. For that, we used plastid markers to understand the phylogenetic relationship between these two species. Then, we used nuclear simple sequence repeat (SSR) polymorphisms to understand the patterns of genetic diversity, differentiation, and genetic structure. For that, we compared the cultivated *C. arabica* plants (Figure 1B) with the ones farmed in northern Mozambique (Niassa) together with the wild relative, *C. racemosa*, that was found during our field surveys (Figure 1C). To complement the SSR study, we investigated coffee genome-wide diversity using single nucleotide polymorphisms (SNP) generated by Genotype-by-Sequencing (GBS) on a reduced sampling set, allowing us to further detect the patterns of diversity and functional pathways involved, as well as to test possible differences with SSR markers. This is the first study that reveals the underlying genomic mechanisms explaining the different adaptation abilities of the cultivated *C. arabica* and the wild relative, *C. racemosa*.

## 2. Results

### 2.1. Plastid Relationships among Coffea Samples

**Figure 2:**
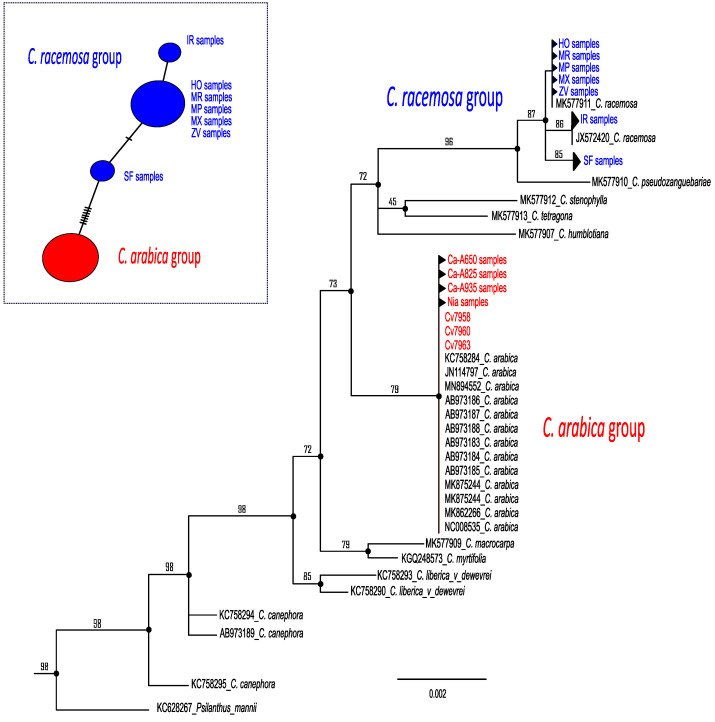
Plastid RaxML tree obtained for *Coffea*. Color codes indicate the two main groups of species: *Coffea arabica* (red) and *Coffea racemosa* (blue) included in this study. Bootstrap values higher than 50% are indicated above branches. Black codes indicate NCBI numbers and species retrieved from GenBank. Insert on the top right indicates the haplotype network using a median-joining method including only *Coffea* sampled for this study.

Two main maternal lineages were retrieved in phylogenetic analyses: one cluster containing all the samples of *C. arabica,* and the other all samples of *C. racemosa* (Figure 2). The cluster containing all *C. arabica* cultivars was phylogenetically apart from all *C. racemosa*. In contrast, three subclusters were retrieved within the *C. racemosa* lineage (Figure 2).

### 2.2. Genetic Diversity in C. arabica and the Wild Relative C. racemosa

Based on nuclear microsatellites, a total of 101 alleles were found among all samples: 56 in the set of *C. racemosa* samples and 41 in *C. arabica* samples or 62 when including also the three cultivars of *C. arabica* from the group Catimor. The average number of alleles and the levels of observed and expected heterozygosity were always lower in *C. arabica* than in *C. racemosa* (Table 1). The mean Shannon Information Index (I) varied from 0.459 among *C. arabica* to 0.905 in *C. racemosa* and was particularly low in the *C. arabica* cultivars of the Gorongosa Agroforestry System (CaAFS) (0.268). The fixation index was negative in all *C. racemosa* accessions, as well as in the *C. arabica* cultivars from Niassa, while the cultivars from the CaAFS and CIFC collection showed positive values of fixation (Table 1). Estimates of genetic diversity based on SNPs revealed extremely low genetic diversity in *C. arabica* (Ho = 1.1± 0.02; He = 2.6 ± 0.9) when compared with *C. racemosa* samples (Ho = 2.9 ± 0.4; He = 3.1 ± 0.12). 

### 2.3. Genetic Structure

**Figure 3:**
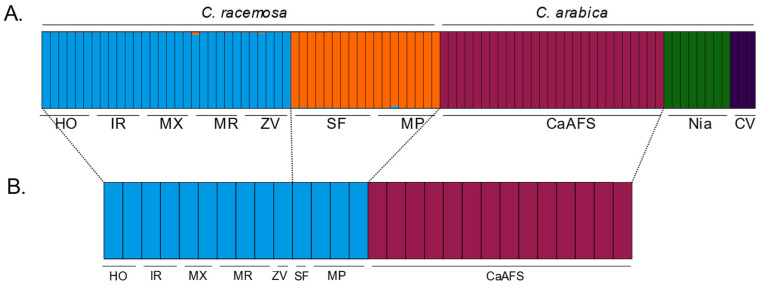
Genetic structure of *Coffea racemosa* and *Coffea arabica*. Genetic clusters are based on the best assignment group found for SSRs (**A**; *K* = 5) and GBS markers (**B**; *K* = 2). Colors indicate an assignment probability, according to different genetic clusters. Each sample is represented by a vertical bar. HO: Homoine, IR: Inharrime, MX: Maxixe; MR: Morrumbene; ZV: Zavala (all from Inhambane Province); MP: Maputo Province; SF: Sofala Province; CaAFS: Gorongosa (Sofala Province); Nia: Niassa Province; CV: CIFC cultivars.

Microsatellite data based on SSRs revealed a total of five genetic clusters among all samples, based on the highest LnP(D) and ΔK values obtained in STRUCTURE HARVESTER (Figure S1). The different genetic membership retrieved divided *C. racemosa* samples from Sofala and Maputo vs. Inhambane (HO, IR, MX, MR, and ZV) provinces (Figure 3A). In *C. arabica*, genetic memberships divided samples from the Gorongosa CaAFS, Niassa (Nia), and the three cultivars from CIFC included in this study (CV) (Figure 3A). However, GBS data retrieved one single genetic membership per species, which segregated *C. racemosa* from *C. arabica* samples (Figure 3B).

No genetic admixture was detected between samples, either using SSRs or GBS data. Linkage disequilibrium (LD) was overall low in *C. racemosa* but significantly higher in *C. arabica* (Figure S2). In both species, LD values did not change significantly (*p* > 0.05) with the increasing physical distance of SNPs.

**Figure 4:**
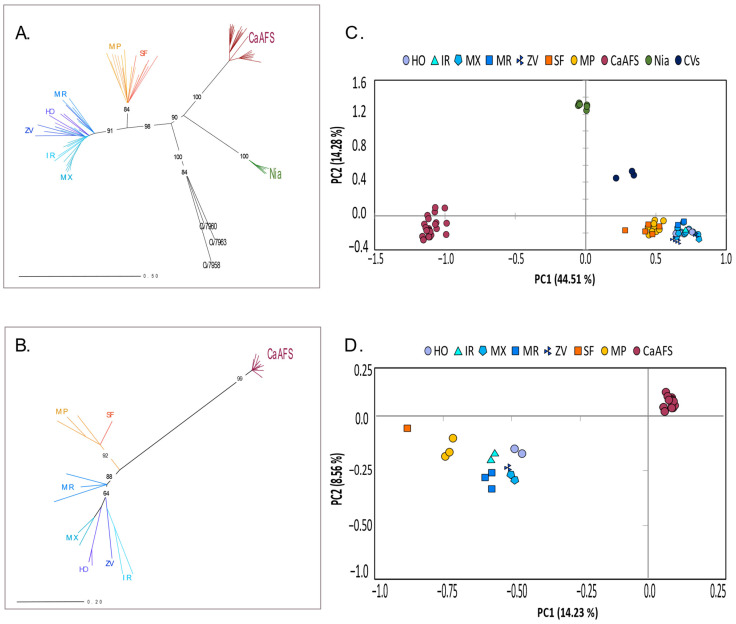
Genetic relationships among *Coffea racemosa* and *Coffea arabica* samples based on Nei’s Genetic Distance. Unrooted Neighbor-Joining (NJ) tree showing relationships among the sampled individuals using the scored nSSRs markers (**A**) and GBS data (**B**). Numbers associated with branches indicate bootstrap values >50 based on 1000 replications. Principal Coordinate Analysis (PCA) scatterplots using the scored nSSRs markers (**C**) and GBS data (**D**). The percentage of explained variance of each axis is given in parentheses. HO: Homoine, IR: Inharrime, MX: Maxixe; MR: Morrumbene; ZV: Zavala (all from Inhambane Province); MP: Maputo Province; SF: Sofala Province; CaAFS: Gorongosa (Sofala Province); Nia: Niassa Province; CV: CIFC cultivars.

Results were generally compatible with the topology of NJ trees and the PCA patterns, which also isolated *C. racemosa* from *C. arabica* samples (Figure 4). It is worth highlighting that Sofala and Maputo are segregated from Inhambane in STRUCTURE, which can also be observed in the NJ tree and PCA from SSR data (Figure 4A,C) but are not well discriminated using the GBS data (Figure 4B,D).

### 2.4. Genetic Differentiation between Species and Sites

Overall, genetic differentiation was significantly high (AMOVA FST = 0.5044, PHI = 0.673, *p* < 0.001). The variance was equally attributed among the *K* = 5 groups found by STRUCTURE (50.45% and 67.32%), and within sites (49.55%; 32.67%) based on SSR and GBS data, respectively. 

**Figure 5:**
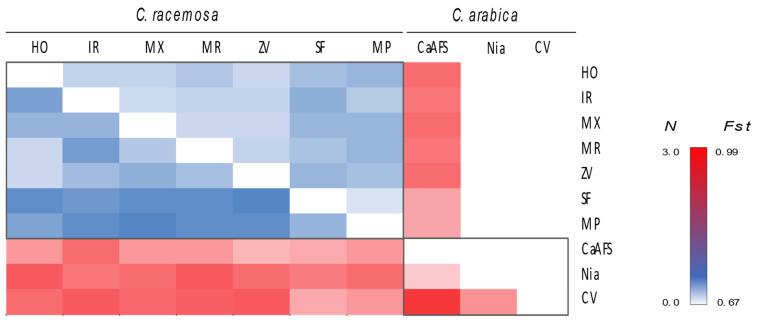
Pairwise differentiation between sites based on Nei’s Genetic Distance using SSRs (below diagonal) and sites differentiation coefficient (Fst) values from GBS (above diagonal) in *Coffea racemosa* and *Coffea arabica*. HO: Homoine, IR: Inharrime, MX: Maxixe; MR: Morrumbene; ZV: Zavala (all from Inhambane Province); MP: Maputo Province; SF: Sofala Province; CaAFS: Gorongosa (Sofala Province); Nia: Niassa Province; CV: CIFC cultivars.

A large genetic divergence was found between *C. arabica* and *C. racemosa* either using pairwise genetic differences of SSRs based on Nei’s Genetic Distance or using Fst values from GBS (Figure 5). It is worth mentioning the large range of divergence found between *C. arabica* cultivars from Gorongosa and the ones collected in Niassa, and even with the CIFC cultivars, where the highest level of divergence was found. Genetic distances were lower between the cultivars of *C. racemosa* sampled in Sofala and Maputo than the ones from Inhambane (HO, IR, MX, MR, and ZV), supporting the previously reported results of genetic structure.

### 2.5. Annotation and Functional Pathways of SNPs

**Figure 6:**
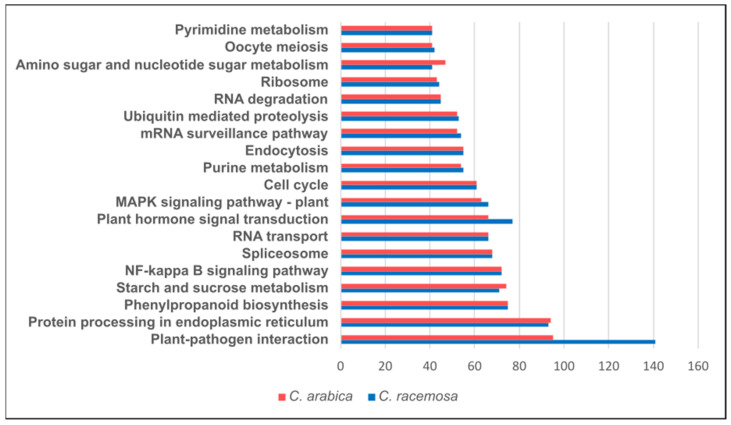
Top KEGG pathways involved among *Coffea arabica* and *Coffea racemosa*.

Sequencing yielded a total of 170,720,052 raw reads, which were reduced to 51,115,669 after quality filtering (Table S6). Overall, an average of 79% of cleaned reads were mapped to the reference genome. A total of 3,058,824 SNPs were found, including 1,461,205 intergenic SNPs, 185,956 intronic SNPs, 115,488 exonic SNPs, 7908 SNPs in splice site, 772,163 upstream, 449,139 downstream, 39,278 in UTR3, and 27,687 in UTR5 (Figure S2). In both species, SNPs were involved in 191 KEGG pathways (Table S2) being top-regulated: ‘*Plant–pathogen interaction*’, ‘*Protein processing in endoplasmic reticulum*’, and ‘*Phenylpropanoid biosynthesis*’ (Figure 6). Only three pathways showed significant differences between the two species: the ‘*Plant–pathogen interaction*’ (F_2,1_ = 25.892, *p* < 0.05) and the ‘*Plant hormone signal transduction*’ (F_2,21_ = 26.034, *p* < 0.05) had more SNPs involved in *C. racemosa* than in *C. arabica* while ‘*Amino sugar and nucleotide sugar metabolism*’ was higher in *C. arabica* than in *C. racemosa* (F_2,23_ = 22.056, *p* < 0.05; Figure 6). Interestingly, SNPs linked with ‘*Caffeine metabolism*’ (Table S2) showed no significant differences between the two cultivars (F_2,23_ = 1.741, *p* > 0.05).

## 3. Discussion

### 3.1. Assessment of Coffea Species in Southern and Central Mozambique Using Plastid Markers

Despite the fact that other wild relatives are supposed to occur in Mozambique such as *C. zanguebariae*, for which herbarium data suggested a sparse distribution in these regions [33,34,35,36], our field expeditions (Figure 1) found only one wild *Coffea* species, *C. racemosa* (known also as Inhambane coffee). Molecular analysis based on plastid markers congruently found two main maternal lineages, splitting this species from *C. arabica* (Figure 2). The cluster grouping *C. arabica* cultivars was phylogenetically apart from *C. racemosa*, supporting a single maternal origin scenario for each species [37]. In contrast, three subclusters were retrieved within the *C. racemosa* lineage, suggesting different origins for this species in Mozambique (Figure 2). 

One explanation for the absence of other wild relatives in these areas could be the genetic drift of *C. zanguebariae* from southern and central Mozambique due to environmental and anthropogenic pressure. Indeed, during our expeditions, we could not validate many historical herbaria locations (personal observations). This was not unexpected and might be interconnected with the fact that (i) Mozambique is among the most disaster-prone countries on a global scale [38], and has gone through a series of natural shocks over the last decades, e.g., the flooding of 2000 and 2017, the Earthquake of 2006, the cyclone Favio in 2007, Idai and Kenneth in 2019, the storm Dando in 2012, or the current tropical storm Freddy that hazards the country as we write this article [39,40]; (ii) the related resettlements of local communities; and (iii) the dynamics of land use and land cover [41]. The second possibility is that the species’ identity has been mistaken in the past. *Coffea racemosa* has been reported as endemic to southern and central Mozambique, distributed across coastal and riverine forests as well as deciduous woodlands and bushlands (0 to 500 m above sea level—a.s.l.), while *C. zanguebariae* was considered endemic to northern Mozambique, distributed across dry deciduous forests and riverine and coastal thickets (0 to 350 m a.s.l.) [33,35]. On the other hand, despite the taxonomic advances to discriminate the two species, *C. racemosa* and *C. zanguebariae* are in fact so similar that they have often been believed to be the same, and only recently, DNA markers allowed accurate species discrimination [19]. It is, thus, possible that many records have misidentified these species although further field expeditions should be done in the north of Mozambique.

### 3.2. Low Genetic Diversity in Coffea arabica in Comparison with the Wild Relative C. racemosa

*Coffea arabica* presented very low levels of genetic diversity in comparison with *C. racemosa* (Table 1). In our study, the mean number of alleles (Na) and effective alleles (Ne) was consistently below two in the cultivars from the three provenances, Gorongosa, Niassa, and CIFC. This value is much lower than those reported in other studies based on SSR markers, which ranged from ca. 3 to 6 [42,43,44,45]. However, the observed heterozygosity (Ho) and expected heterozygosity (He) values were within the expected range considering the low genetic values usually reported in microsatellite studies of *C. arabica* [45,46]. In addition, our estimates of genetic diversity based on SNPs also revealed extremely low values in *C. arabica* when compared with *C. racemosa*. In fact, the overall congruent results found between SSR and SNP data suggest that these independent markers can detect similar patterns of genetic diversity. However, between the two markers, SSRs remain the most cost-effective and rapid marker being widely used in most genetic population studies. 

The low levels of heterozygosity are likely due to the autogamous nature of *C. arabica* [45,47,48,49] and the single polyploidization event at the origin of the tetraploid genome of this species, which was probably narrowed further in some cultivars of this species [14]. Indeed, the Shannon diversity index (I) of all *C. arabica* cultivars used in this study was very low (0.48 on average), supporting the genetic bottleneck hypothesis in commercial Arabica varieties [44]. In contrast, the consistently high diversity levels observed in *C. racemosa* are likely due to the allogamy of the species [50]. These genetic diversity results were comparable to those reported in other tropical trees, such as *Warburgia salutaris* from southern Mozambique [51] or *Ocotea rotundata* from the northern Andean forests [52], suggesting that *C. racemosa* retains high levels of genetic diversity, especially when compared with *C. arabica*. 

The absence of gene flow between the two species would explain the finding of different genetic clusters (and the absence of genetic admixture) that segregated all *C. racemosa* from the *C. arabica* sample, either when considering STRUCTURE results (Figure 3), the topology of NJ trees, or PCA patterns (Figure 4). The large genetic divergence found between *C. arabica* and *C. racemosa* is also supported by the pairwise genetic differences of SSRs based on Nei’s Genetic Distance and the Fst values from GBS (Figure 5). It is also worth mentioning the large range of divergence found between *C. arabica* cultivars from Gorongosa and the ones implemented in Niassa, and even with the CIFC cultivars, where the highest level of divergence was found. Gene flow that usually results from pollen and seed migration plays a significant role in preventing genetic differentiation among populations while contributing to the conservation of genetic diversity [53]. The autogamous nature of the cultivated *C. arabica* contributes to such differentiation and is a concern in light of environmental changes. In contrast, pollination by birds or insects and the dispersion of seeds are likely to occur in *C. racemosa*, contributing to the patterns of genetic diversity and structure found in this study. Additionally, although hybrids between the diploid wild *C. racemosa* and the tetraploid cultivated *C. arabica* would be possible, hybrid triploid plants are expected to be infertile (but see [54]). 

As a wild relative of coffee and despite the important role that *C. racemosa* might have to implement sustainable changes in the coffee sector, fundamental basic data, such as the type of breeding system, and the type of pollinators and dispersers involved in this species are unknown. This is particularly significant as we found differences in the functional pathways of these two species that could reflect differences in the tolerance to environmental stresses (Figure 6). For instance, the ‘*Plant–pathogen interaction*’ and ‘*Plant hormone signal transduction*’ were significantly more represented in *C. racemosa*, suggesting that the signaling pathways related to stress tolerance are more robust in this species [55]. On the other hand, *C. arabica* was more enriched in SNPs involved in amino sugar and nucleotide sugar metabolism than *C. racemosa*. Some enzyme proteins in these pathways are also involved in stress response in plants and thus, a greater number of genes from these pathways may be redundant, as they are important to maintain pivotal functions, including cell wall synthesis and cell repair processes (e.g., associated with pectin synthesis) [53,56]. Interestingly, SNPs linked with the ‘*Caffeine metabolism’* showed no significant differences between the two cultivars (Table S2), even though *C. racemosa* is sought as a “naturally decaffeinated” bean due to its low levels of caffeine [19,57]. Based on local records, this species is thought to produce an aromatic drink with low caffeine levels [58]. This highlights the need for more studies on the functional traits of *C. racemosa*, namely the quality of its beans.

### 3.3. Implications for the Management of the Gorongosa Agroforestry System

The use of AFS in Gorongosa Nacional Park is seen as a promising and compatible approach to help adapt to climate change while reconciling biodiversity conservation and local development [10,26,59]. The fact that coffee originates from high-altitude forest regions and can develop in shady areas [36], together with the historical context of coffee in Mozambique, were the main reasons for the implantation of this system in the Gorongosa Mountains. However, our results showed very low genetic diversity values in *C. arabica* plants used in the AFS, which could affect the long-term sustainability of this system. In this context, the introduction of new Arabica cultivars in the Gorongosa AFS would be an asset, given the low genetic diversity of the implanted cultivar. More efforts involving coffee producers should be developed to create awareness of the importance of conserving *C. racemosa*. Additionally, attempts to introduce *C. racemosa* and other crop-wild relatives into the value chain should be placed on the agenda. Wild coffee species are already being farmed in Kwa Zulu Natal in South Africa (Hluhluwe) [19], and although yields are low when compared with the widely used *C. arabica* and *C. canephora*, their specific attributes regarding environmental stress tolerance and flavor could be useful for new blends [19]. Recognizing the global socioeconomic importance of coffee, particularly in many developing countries that largely depend on this commodity, it is vital to innovate the coffee value chain, introducing more resilient species, increasing genomic diversity, and adopting climate-smart practices.

## 4. Materials and Methods

### 4.1. Plant Sampling and DNA Isolation

Thirty-five samples of *C. arabica* (27 from the Gorongosa Mountain, Sofala Province; and eight from Niassa Province) and 48 wild relatives collected in three provinces from central and southern Mozambique (Maputo, Inhambane, and Sofala) (Figure 2) were included in the analysis. This region gathered most of the historical collections recorded for *Coffea* in Mozambique. The study also included three additional commercial genotypes from Centro de Investigação das Ferrugens do Cafeeiro (CIFC), totaling 86 samples. The main variety of *C. arabica* cultivated in the Gorongosa Mountain is a commercial variety imported from Zimbabwe due to similar agroecological conditions in both countries. The cultivar is labeled Costa Rica (CR) and it is claimed to be tolerant to coffee leaf rust and coffee berry disease. The CIFC cultivars are certified hybrids of the Catimor group (CR-95). *Coffea arabica* is a tetraploid species with 2n = 44 chromosomes that usually behave genetically as diploid [60] while *C. racemosa* is a diploid species with 2n = 22 chromosomes [61]. In each site, 6 to 10 individuals were randomly collected with a minimum sampling distance of 10 m. Samples, locations, and geographic coordinates are shown in Table S1. 

Fresh leaves were collected for each sample, dried on silica gel, and stored at −80 °C until DNA was extracted. Total genomic DNA was extracted from 100 mg of ground leaves using the InnuPrep Plant DNA kit (Analytik Jena Innuscreen GmbH, Jena, Germany) according to the manufacturer’s protocol. Mean yield and purity were evaluated spectrophotometrically by readings of OD230, OD260, and OD280 (Nanodrop 2000, Thermo Fisher Scientific, Waltham, MA, USA) and visualized by 1% agarose gel electrophoresis under UV light.

### 4.2. Plastid Barcode Sequencing

Two barcode organelle regions (*rbcL* and *matK*) previously used in *Coffea* (Table S3) were first amplified to detect the degree of haplotype variation using the 86 samples. Polymerase chain reactions (PCR) were performed in 20 µL reactions using Biotaq DNA polymerase (Bioline, London, UK), 2X reaction buffer (Bioline, London, UK), 1 μM forward and reverse primers, 2 mM MgCl_2_, and dNTPs 0.8 mM (Promega, Maddison, WI, USA), 0.2 U Taq Meridian Bioscience (MI, Italy), 0.28 mg/mL BSA, and 40 ng μL^−1^ of genomic DNA. Cycle sequencing reactions were carried out using the Bio-Rad PCR System MyCycler™ thermocycler. The PCR program for *rbcL* consisted of 4 min at 94 °C followed by 35 cycles of 30 s at 94 °C, 1 min at 55 °C, and 1 min at 72 °C, with a final extension of 10 min at 72 °C. For *matK*, amplifications consisted of 5 min at 95 °C followed by 40 cycles of 30 sec at 95 °C, 30 s at 52 °C, and 1 min at 72 °C, with a final extension of 5 min at 72 °C. Amplified products were purified using QIAquick purification columns (QIAgen, Madrid, Spain), as described in the manufacturer’s protocol, and sent for sequencing (Macrogen, Madrid, Spain). Consensus alignments for each gene were created in Geneious v.11.1.5 (Biomatters, Ltd., Auckland, New Zealand) using the MAFFT alignment algorithm v.7.450 [62] and manually checked. A phylogeny based on a maximum likelihood (ML) analysis was performed using the two plastid genes concatenated into a single matrix. Additionally, data from other representatives of the same species studied here, as well as representative outgroup taxa, were extracted from the NCBI database (Table S4). The best-fitting nucleotide substitution model was estimated using jModelTest2 v. 2.1.6 [63] (GTR) and used as input for RAxML v.8.2.12 with 1.000 bootstrap iterations [64]. In addition, genealogical haplotype relationships of the collected samples were inferred using the median-joining method in Popart v1.7 [65].

### 4.3. Single-Sequence Polymorphic Repeats

The 86 samples were genotyped at 14 nuclear single-sequence polymorphic repeats (SSRs) previously developed for *Coffea* (Table S5). Based on the initial research, we selected these 14 SSRs markers as they produced robust and highly polymorphic amplified bands across all collections of the samples under study. Amplifications were performed in 20 μL reaction volume containing 1 μM forward and reverse primers, 2X Buffer Meridian Bioscience (MI, Italy), 0.5 U of TAQ Meridian Bioscience (MI, Italy), and 40 ng μL−1 of genomic DNA on a Bio-Rad PCR System MyCycler™ thermocycler. Allele sizes were determined using Peak Scanner version 1.0 (Life Technologies, Carlsbad, CA, USA) and revised manually. 

### 4.4. GBS Library Preparation, Sequencing, and SNP Calling

Genomic DNA (0.3~0.6 µg) of a subset of *Coffea* samples (28 total; Table S1) was double-digested using 10 μL of the restriction enzymes EcoRI and Mse I for 5 h at 37 °C, then 20 min at 65 °C, and final incubation at 12 °C. The resulting digested fragments were cleaned and subsequently quantified using agarose gel electrophoresis and the Qubit^®^2.0 fluorometer. Digested fragments were ligated to EcoR I and Mse I adapters containing sample-specific barcodes with T4 DNA ligase (NEB) for 4 h at 16 °C, then 20 min at 65 °C, and final incubation at 12 °C. Individually barcoded samples were cleaned and size-selected (350–500 bp) using agarose gel. After dilution to 1 ng µL^−1^, the Agilent^®^2100 bioanalyzer was used to assess insert size. Each library was then PCR-amplified to the desired concentration and paired-end sequenced on an Illumina^®^HiSeq PE150.

FastQC [66] was used to remove adapters, and low-quality reads, e.g., uncertain nucleotides > 10% and base quality < 5 in more than 50% of either read, consistent with an error rate < 0.1%. Assembled reads were mapped against the reference genome of *C. arabica* downloaded from the NCBI (https://www.ncbi.nlm.nih.gov/assembly/GCF_003713225.1, accessed on 4 April 2021) using BWA version 0.7.16 [67] with the default parameters. The resulting individual files were converted into BAM files with SAMtools version 1.16.1 [68], removing duplicate reads. Sequencing yielded a total of 170,720,052 raw reads, which were reduced to 51,115,669 after quality filtering (Table S6). Overall, an average of 79% of cleaned reads were mapped to the reference genome. Calling of variants (SNPs) was performed for the 28 sequenced *Coffea* samples using GATK 4.2.6.1 [69] with base quality score recalibration, indel realignment, duplicate removal, and performed SNP and INDEL discovery. Genotyping across samples was performed simultaneously using standard hard filtering parameters or variant quality score recalibration according to GATK Best Practices recommendations [70]. Filtering of SNPs included those with a sequencing depth of 3 to 50 for each sample and an average quality > 20. To exclude SNP calling errors caused by incorrect mapping or indels, two adjacent SNPs separated by <5 bp were not called. A total of 3,058,824 SNPs were found, including 1,461,205 intergenic SNPs, 185,956 intronic SNPs, 115,488 exonic SNPs, 7908 SNPs in splice site, 772,163 upstream, 449,139 downstream, 39,278 in UTR3, and 27,687 in UTR5. The location and annotation of SNPs were based on the data retrieved from the reference genome of *C. arabica* as mentioned above. Associated genes were mapped to the KEGG [71] pathway and were examined if they were enriched in particular pathways based on the hypergeometric distribution test. Fisher’s exact test was used to identify pathways significantly enriched (*p* < 0.05) with *Coffea* genes.

### 4.5. Genetic Diversity, Structure, and Differentiation 

Since genetic data of the two species were diploidized (e.g., only a maximum of two alleles were found), we used the Bayesian program STRUCTURE v.2.3.4 [72] to test whether any discrete genetic structure existed among samples and species. The analysis was performed assuming *K* = 1 to *K* = 10 genetic clusters (*K*), with 10 repetitions per *K*. Models were run assuming ancestral admixture and correlated allele frequencies using run lengths of 200,000 interactions for each K after 50,000 burn-in steps. The optimum *K* value was determined using STRUCTURE HARVESTER [73], which identifies the optimal *K* based on both the posterior probability of the data for a given K and the ∆K [74]. The results of the replicates at the best-fit *K* were then post-processed using CLUMPAK [75]. To visualize the genetic structure, a Principal Components Analysis (PCA) and a Neighbor-Joining (NJ) tree were constructed with 10,000 bootstraps in the adegenet R package [76]. Differentiation between sites was analyzed by conducting an analysis of molecular variance (AMOVA) using Arlequin 3.5.2.2 [77]. This approach is derived from the analysis of the variance framework based on Wright’s fixation indices defined by [78]. Pairwise differentiation between species and sites was also computed based on Nei’s Genetic Distance and the coefficient of differentiation (Fst). Genetic diversity was assessed by calculating the number of alleles (Na), observed heterozygosity (Ho), expected heterozygosity (He), and fixation index (F), using diveRsity [79] and PopPr R packages [80]. We also calculated linkage disequilibrium (LD) pruning the SNPs using Plink v1.9 [81] with a window of 50 SNPs and a step size of five makers. PLINK was used to measure pairwise LD between multi-SNPs based on the allele frequency correlations. The LD decay plot was drawn using R (http://www.R-project.org/, accessed on 22 June 2021). Functional annotation of the SNPs was defined using the Blast2GO V5.0 tool [82] (E-value ≥ 1 × 10^−5^) implemented in the KEGG database [71].

## 5. Conclusions

Here, we show for the first time how genetic diversity is needed to assure sustainable agriculture practices. Even though the implementation of AFSs is of interest since they can offset deforestation in tropical environments, while increasing biodiversity, productivity, social profitability, and environmental stewardship, guaranteeing the genetic diversity of the species/cultivars is an essential condition to ensure the long-term sustainability of AFSs. In this sense, the introduction of crop wild relatives in coffee AFSs provides an opportunity to increase the productivity and resilience of agricultural systems as they contain useful genetic diversity, which as reported here is not present in cultivated Arabica coffee.

## Figures and Tables

**Table 1:** Estimates of genetic diversity based on SSRs for *Coffea arabica* and *Coffea racemosa*. HO: Homoine, IR: Inharrime, MX: Maxixe; MR: Morrumbene; ZV: Zavala (all from Inhambane Province); MP: Maputo Province; SF: Sofala Province; CaAFS: Gorongosa (Sofala Province); Nia: Niassa Province; CV: CIFC cultivars; Na: number of alleles; Ne: Number of effective alleles; I: Shannon’s Information Index; Ho: observed heterozygosity; He: expected heterozygosity; F = fixation index.

Species	Sites	Na	Ne	I	Ho	He	F
*C. racemosa*	HO	3.00 ± 0.21	2.59 ± 0.17	0.98 ± 0.06	0.93 ± 0.03	0.59 ± 0.02	−0.58 ± 0.07
	IR	2.71 ± 0.27	2.33 ± 0.19	0.85 ± 0.09	0.89 ± 0.07	0.53 ± 0.05	−0.71 ± 0.08
	MX	2.71 ± 0.19	2.33 ± 0.14	0.87 ± 0.07	0.89 ± 0.07	0.54 ± 0.04	−0.63 ± 0.11
	MR	2.86 ± 0.18	2.34 ± 0.13	0.89 ± 0.07	0.86 ± 0.07	0.55 ± 0.03	−0.55 ± 0.10
	ZV	3.00 ± 0.26	2.47 ± 0.18	0.94 ± 0.07	0.89 ± 0.05	0.57 ± 0.03	−0.56 ± 0.08
	SF	2.93 ± 0.22	2.45 ± 0.20	0.91 ± 0.08	0.89 ± 0.06	0.55 ± 0.04	−0.59 ± 0.08
	MP	2.92 ± 0.29	2.36 ± 0.15	0.90 ± 0.07	0.95 ± 0.03	0.56 ± 0.02	−0.72 ± 0.08
	average	2.88 ± 0.09	2.41 ± 0.06	0.91 ± 0.03	0.90 ± 0.02	0.56 ± 0.01	−0.62 ± 0.03
*C. arabica*	CaAFS	1.93 ± 0.17	1.26 ± 0.10	0.27 ± 0.06	0.16 ± 0.07	0.16 ± 0.05	0.02 ± 0.11
	Nia	1.88 ± 0.16	1.80 ± 0.13	0.56 ± 0.09	0.69 ± 0.12	0.39 ± 0.06	−0.80 ± 0.11
	CV	1.79 ± 0.24	1.73 ± 0.23	0.45 ± 0.13	0.05 ± 0.03	0.29 ± 0.08	0.83 ± 0.08
	average	1.87 ± 0.08	1.68 ± 0.07	0.48 ± 0.04	0.46 ± 0.05	0.39 ± 0.03	−0.40 ± 0.09
All samples		2.46 ± 0.08	2.10 ± 0.054	0.73 ± 0.03	0.72 ± 0.03	0.46 ± 0.01	0.55 ± 0.04

## Data Availability

Nucleotide sequences produced in this study are available in NCBI GenBank under OP207780—OP207865 (*rbcL*) and OP320952-OP321037 (*matK*). Raw data reads are deposited at NCBI SRA database under Bioproject PRJNA947603.

## Supplementary Materials

The following supporting information can be downloaded at: https://www.mdpi.com/article/10.3390/plants12102044/s1. Figure S1: Mean Ln probability of data and Delta K based on Evanno’s ad hoc statistic obtained by STRUCTURE HARVESTER. Figure S2: Scatter plot of linkage disequilibrium decay (r^2^) against the genetic distance for pairs of linked SNPs considering *Coffea racemosa* and *Coffea arabica*. Table S1: Sampling information of *Coffea arabica and Coffea racemosa* sorted by geographical area including the individuals used for cpDNA (*rbcL* and *matk*), SSRs and SNPs analyses. Genbank numbers indicate the new sequences obtained in this study. Table S2: KEGG pathways found in *Coffea racemosa* and *Coffea arabica*. Table S3: Plastid primers used to amplify the *Coffea* samples. Table S4: Taxa retrieved from NCBI and used in the phylogenetic analyses. Table S5: SSRs primers used to amplify the *Coffea* samples. Table S6: Summary of sequencing and mapping of reads from *Coffea arabica* and *C. racemosa* samples.
