# Supplementary material for: Genomic Evaluation of Coffea arabica and Its Wild Relative Coffea racemosa in Mozambique: Settling Resilience Keys for the Coffee Crop in the Context of Climate Change
Source: Plants (Basel). 2023 May 20;12(10):2044. doi: 10.3390/plants12102044 (PMC10220690; doi:10.3390/plants12102044)
Supplement: Supplementary file 1 [file plants-12-02044-s001.zip › plants-2305959-supplementary.pdf]

Supporting Information Files

Figure S1. Mean Ln probability of data and Delta K based on Evanno's ad hoc statistic obtained by STRUCTURE HARVESTER.

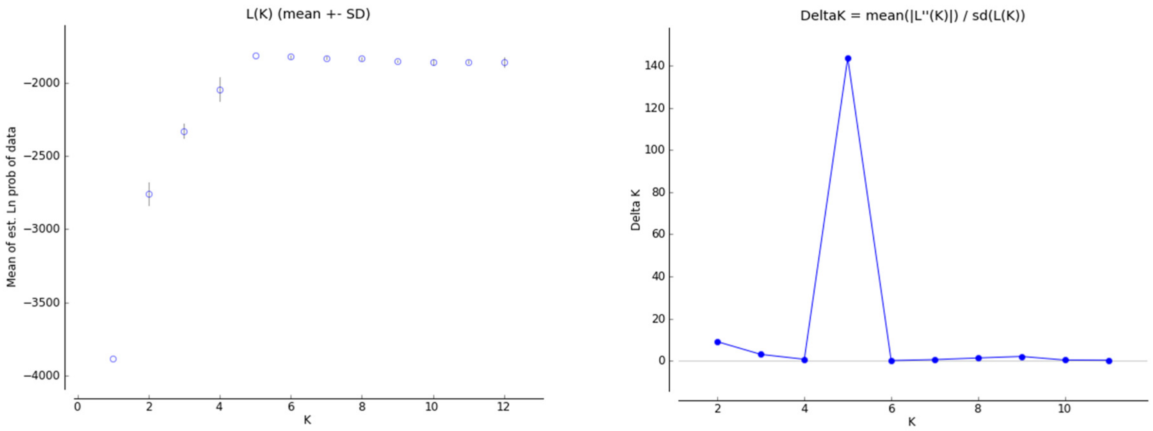

Figure S2. Scatter plot of Linkage disequilibrium decay (r^2) against the genetic distance for pairs of linked SNPs considering *Coffea racemosa* and *Coffea arabica*.

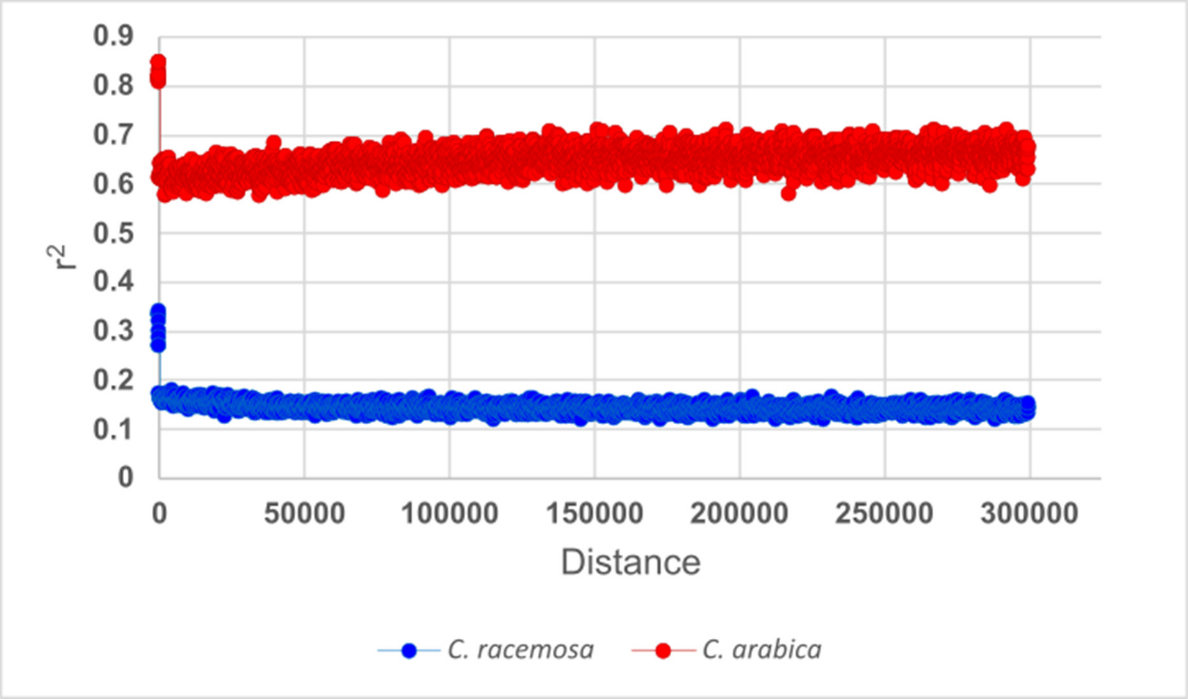

Table S1. Sampling information of *Coffea arabica* and *Coffea racemosa* sorted by geographical area including the individuals used for cpDNA (*rbcL* and *matk*), SSRs and SNPs analyses. Genbank numbers indicate the new sequences obtained in this study.

ID	Species	Province	Population	Latitude	Longitude	*rbcL*	*matK*	SSRs	SNPs
**IR01**	*Coffea racemosa*	Inhambane	Inharrime	-24,30259	34,90368	OP207780	OP320952	x	x
**IR02**	*Coffea racemosa*	Inhambane	Inharrime	-24,47284	35,03484	OP207781	OP320953	x	x
**IR03**	*Coffea racemosa*	Inhambane	Inharrime	-24,47282	35,03481	OP207782	OP320954	x	
**IR04**	*Coffea racemosa*	Inhambane	Inharrime	-24,47329	35,02921	OP207783	OP320955	x	
**IR05**	*Coffea racemosa*	Inhambane	Inharrime	-24,47431	35,02438	OP207784	OP320956	x	
**IR06**	*Coffea racemosa*	Inhambane	Inharrime	-24,45185	35,01889	OP207785	OP320957	x	
**ZV01**	*Coffea racemosa*	Inhambane	Zavala	-24,50669	34,99925	OP207786	OP320958	x	x
**ZV02**	*Coffea racemosa*	Inhambane	Zavala	-24,50669	34,99922	OP207787	OP320959	x	
**ZV03**	*Coffea racemosa*	Inhambane	Zavala	-24,54388	34,88284	OP207788	OP320960	x	
**ZV05**	*Coffea racemosa*	Inhambane	Zavala	-24,68345	34,54291	OP207789	OP320961	x	
**ZV06**	*Coffea racemosa*	Inhambane	Zavala	-24,68415	34,54329	OP207790	OP320962	x	
**ZV07**	*Coffea racemosa*	Inhambane	Zavala	-24,68736	34,54589	OP207791	OP320963	x	
**Ho01**	*Coffea racemosa*	Inhambane	Homoine	-23,87354	35,24534	OP207792	OP320964	x	
**HO03**	*Coffea racemosa*	Inhambane	Homoine	-23,87354	35,24826	OP207793	OP320965	x	x
**HO04**	*Coffea racemosa*	Inhambane	Homoine	-23,89639	35,15764	OP207794	OP320966	x	x
**HO05**	*Coffea racemosa*	Inhambane	Homoine	-23,89639	35,15766	OP207795	OP320967	x	
**HO06**	*Coffea racemosa*	Inhambane	Homoine	-23,88346	35,15156	OP207796	OP320968	x	
**HO07**	*Coffea racemosa*	Inhambane	Homoine	-23,88332	35,15153	OP207797	OP320969	x	
**MX01**	*Coffea racemosa*	Inhambane	Maxixe	-23,86021	35,33956	OP207798	OP320970	x	
**MX02**	*Coffea racemosa*	Inhambane	Maxixe	-23,83998	35,34383	OP207799	OP320971	x	
**MX03**	*Coffea racemosa*	Inhambane	Maxixe	-23,82475	35,34215	OP207800	OP320972	x	x
**MX04**	*Coffea racemosa*	Inhambane	Maxixe	-23,82475	35,34218	OP207801	OP320973	x	
**MX05**	*Coffea racemosa*	Inhambane	Maxixe	-23,82457	35,34183	OP207802	OP320974	x	x
**MX08**	*Coffea racemosa*	Inhambane	Maxixe	-23,82476	35,34279	OP207803	OP320975	x	
**MR02**	*Coffea racemosa*	Inhambane	Morrumbene	-23,61939	35,24733	OP207804	OP320976	x	x
**MR03**	*Coffea racemosa*	Inhambane	Morrumbene	-23,61931	35,24752	OP207805	OP320977	x	x
**MR04**	*Coffea racemosa*	Inhambane	Morrumbene	-23,61926	35,24751	OP207806	OP320978	x	
**MR05**	*Coffea racemosa*	Inhambane	Morrumbene	-23,61922	35,24752	OP207807	OP320979	x	

**MR06**	*Coffea racemosa*	Inhambane	Morrumbene	-23,69177	35,31211	OP207808	OP320980	x	
**MP02**	*Coffea racemosa*	Maputo	Matola	-25,92764	32,54849	OP207809	OP320981	x	
**MP03**	*Coffea racemosa*	Maputo	Matola	-25,96622	32,46106	OP207810	OP320982	x	
**MP04**	*Coffea racemosa*	Maputo	Matola	-25,95636	32,45265	OP207811	OP320983	x	x
**MP05**	*Coffea racemosa*	Maputo	Matola	-25,95733	32,45744	OP207812	OP320984	x	x
**MP06**	*Coffea racemosa*	Maputo	Matola	-25,95636	32,45255	OP207813	OP320985	x	
**MP07**	*Coffea racemosa*	Maputo	Matola	-25,95623	32,45265	OP207814	OP320986	x	
**MP08**	*Coffea racemosa*	Maputo	Matola	-25,95636	32,45232	OP207815	OP320987	x	x
**MP09**	*Coffea racemosa*	Maputo	Matola	-25,95625	32,45245	OP207816	OP320988	x	
**MP10**	*Coffea racemosa*	Maputo	Matola	-25,95321	32,45231	OP207817	OP320989	x	
**SF02**	*Coffea racemosa*	Sofala	Gorongosa	-18,56341	34,08541	OP207818	OP320990	x	
**SF05**	*Coffea racemosa*	Sofala	Gorongosa	-19,06242	34,25271	OP207819	OP320991	x	x
**SF06**	*Coffea racemosa*	Sofala	Gorongosa	-18,56271	39,08541	OP207820	OP320992	x	
**SF07**	*Coffea racemosa*	Sofala	Gorongosa	-18,56341	34,09212	OP207821	OP320993	x	
**SF08**	*Coffea racemosa*	Sofala	Gorongosa	-19,00011	34,12044	OP207822	OP320994	x	
**SF17**	*Coffea racemosa*	Sofala	Gorongosa	-18,56234	34,09189	OP207823	OP320995	x	
**SF21**	*Coffea racemosa*	Sofala	Gorongosa	-18,56224	34,09196	OP207824	OP320996	x	
**SF25**	*Coffea racemosa*	Sofala	Gorongosa	-18,56235	34,08568	OP207825	OP320997	x	
**SF29**	*Coffea racemosa*	Sofala	Gorongosa	-18,56199	34,08572	OP207826	OP320998	x	
**SF31**	*Coffea racemosa*	Sofala	Gorongosa	-18,56167	34,08555	OP207827	OP320999	x	
**NIA01**	*Coffea arabica*	Niassa	Maua	-13,44471	36,59212	OP207828	OP321000	x	
**NIA02**	*Coffea arabica*	Niassa	Maua	-13,44471	36,59229	OP207829	OP321001	x	
**NIA03**	*Coffea arabica*	Niassa	Maua	-13,19508	35,14472	OP207830	OP321002	x	
**NIA04**	*Coffea arabica*	Niassa	Maua	-13,19508	35,14485	OP207831	OP321003	x	
**NIA05**	*Coffea arabica*	Niassa	Maua	-12,52288	35,43506	OP207832	OP321004	x	
**NIA06**	*Coffea arabica*	Niassa	Maua	-12,52288	35,43506	OP207833	OP321005	x	
**NIA07**	*Coffea arabica*	Niassa	Maua	-13,20231	35,16513	OP207834	OP321006	x	
**NIA08**	*Coffea arabica*	Niassa	Maua	-13,20232	35,16511	OP207835	OP321007	x	
**Ca-A650-P1**	*C. arabica* cv. Costa Rica 95	Sofala	PNG	-18,51435	34,05135	OP207836	OP321008	x	
**Ca-A650-P2**	*C. arabica* cv. Costa Rica 95	Sofala	PNG	-18,51431	34,05133	OP207837	OP321009	x	
**Ca-A650-P3**	*C. arabica* cv. Costa Rica 95	Sofala	PNG	-18,51439	34,05121	OP207838	OP321010	x	x

**Ca-A650-P6**	*C. arabica* cv. Costa Rica 95	Sofala	PNG	-18,51428	34,05132	OP207839	OP321011	x	x
**Ca-A650-P7**	*C. arabica* cv. Costa Rica 95	Sofala	PNG	-18,51427	34,05128	OP207840	OP321012	x	x
**Ca-A650-P8**	*C. arabica* cv. Costa Rica 95	Sofala	PNG	-18,51436	34,05124	OP207841	OP321013	x	x
**Ca-A650-P11**	*C. arabica* cv. Costa Rica 95	Sofala	PNG	-18,51448	34,05127	OP207842	OP321014	x	x
**Ca-A650-P12**	*C. arabica* cv. Costa Rica 95	Sofala	PNG	-18,51466	34,05122	OP207843	OP321015	x	
**Ca-A650-P13**	*C. arabica* cv. Costa Rica 95	Sofala	PNG	-18,51469	34,05125	OP207844	OP321016	x	
**Ca-A825-P16**	*C. arabica* cv. Costa Rica 95	Sofala	PNG	-18,50061	34,04907	OP207845	OP321017	x	x
**Ca-A825-P17**	*C. arabica* cv. Costa Rica 95	Sofala	PNG	-18,50062	34,04911	OP207846	OP321018	x	
**Ca-A825-P18**	*C. arabica* cv. Costa Rica 95	Sofala	PNG	-18,50073	34,04921	OP207847	OP321019	x	x
**Ca-A825-P21**	*C. arabica* cv. Costa Rica 95	Sofala	PNG	-18,50059	34,04905	OP207848	OP321020	x	
**Ca-A825-P22**	*C. arabica* cv. Costa Rica 95	Sofala	PNG	-18,50062	34,04904	OP207849	OP321021	x	x
**Ca-A825-P23**	*C. arabica* cv. Costa Rica 95	Sofala	PNG	-18,50062	34,04901	OP207850	OP321022	x	x
**Ca-A825-P26**	*C. arabica* cv. Costa Rica 95	Sofala	PNG	-18,50043	34,04907	OP207851	OP321023	x	x
**Ca-A825-P27**	*C. arabica* cv. Costa Rica 95	Sofala	PNG	-18,50043	34,04906	OP207852	OP321024	x	
**Ca-A825-P28**	*C. arabica* cv. Costa Rica 95	Sofala	PNG	-18,50081	34,04949	OP207853	OP321025	x	
**Ca-A935-P31**	*C. arabica* cv. Costa Rica 95	Sofala	PNG	-18,48163	34,04477	OP207854	OP321026	x	
**Ca-A935-P32**	*C. arabica* cv. Costa Rica 95	Sofala	PNG	-18,48162	34,04478	OP207855	OP321027	x	
**Ca-A935-P33**	*C. arabica* cv. Costa Rica 95	Sofala	PNG	-18,48163	34,04475	OP207856	OP321028	x	x
**Ca-A935-P37**	*C. arabica* cv. Costa Rica 95	Sofala	PNG	-18,48158	34,04469	OP207857	OP321029	x	x
**Ca-A935-P38**	*C. arabica* cv. Costa Rica 95	Sofala	PNG	-18,48158	34,04469	OP207858	OP321030	x	x
**Ca-A935-P41**	*C. arabica* cv. Costa Rica 95	Sofala	PNG	-18,48151	34,04467	OP207859	OP321031	x	x
**Ca-A935-P42**	*C. arabica* cv. Costa Rica 95	Sofala	PNG	-18,48151	34,04466	OP207860	OP321032	x	
**Ca-A935-P43**	*C. arabica* cv. Costa Rica 95	Sofala	PNG	-18,48187	34,04402	OP207861	OP321033	x	x
**Ca-A935-P44**	*C. arabica* cv. Costa Rica 95	Sofala	PNG			OP207862	OP321034	x	
**Cv7960**	*C. arabica*		Cultivar			OP207863	OP321035	x	
**Cv7963**	*C. arabica*		Cultivar			OP207864	OP321036	x	
**Cv7958**	*C. arabica*		Cultivar			OP207865	OP321037	x	

Table S2. KEGG pathways found in *Coffea racemosa* and *Coffea arabica*.

**pathways**	***Coffea racemosa***	***Coffea arabica***
Plant-pathogen interaction	141	95
Protein processing in endoplasmic reticulum	93	94
Phenylpropanoid biosynthesis	75	75
Starch and sucrose metabolism	71	74
NF-kappa B signaling pathway	72	72
Spliceosome	68	68
RNA transport	66	66
Plant hormone signal transduction	77	66
MAPK signaling pathway - plant	66	63
Cell cycle	61	61
Purine metabolism	55	54
Endocytosis	55	55
mRNA surveillance pathway	54	52
Ubiquitin mediated proteolysis	53	52
RNA degradation	45	45
Ribosome	44	43
Amino sugar and nucleotide sugar metabolism	41	42
Oocyte meiosis	42	41
Pyrimidine metabolism	41	41
Phosphatidylinositol signaling system	41	41
Autophagy - animal	40	40
Inositol phosphate metabolism	39	39
Glycolysis / Gluconeogenesis	37	37
Ribosome biogenesis in eukaryotes	36	36
Glycine, serine and threonine metabolism	35	35
ABC transporters	35	35
Pentose and glucuronate interconversions	35	34
Tyrosine metabolism	32	32
Aminoacyl-tRNA biosynthesis	32	32

Galactose metabolism	30	30
Glycerophospholipid metabolism	30	30
Carbon fixation in photosynthetic organisms	30	30
Phenylalanine metabolism	29	29
Cyanoamino acid metabolism	29	29
Regulation of actin cytoskeleton	29	29
Alanine, aspartate and glutamate metabolism	28	28
cAMP signaling pathway	28	28
Phospholipase D signaling pathway	28	28
Glutathione metabolism	27	27
Isoquinoline alkaloid biosynthesis	27	27
Nucleotide excision repair	27	27
Fanconi anemia pathway	27	27
Sphingolipid signaling pathway	27	27
Carotenoid biosynthesis	26	26
Quorum sensing	26	26
Cellular senescence	26	26
Cysteine and methionine metabolism	25	25
Oxidative phosphorylation	24	24
Glyoxylate and dicarboxylate metabolism	24	24
One carbon pool by folate	24	24
Stilbenoid, diarylheptanoid and gingerol biosynthesis	24	24
Ras signaling pathway	24	24
AMPK signaling pathway	24	24
Glycerolipid metabolism	23	23
Ascorbate and aldarate metabolism	22	22
Arginine biosynthesis	22	22
Pyruvate metabolism	22	22
Sesquiterpenoid and triterpenoid biosynthesis	22	22
Phagosome	22	22
Tight junction	22	22
Fructose and mannose metabolism	21	21

Phenylalanine, tyrosine and tryptophan biosynthesis	21	21
Nitrogen metabolism	21	21
DNA replication	21	21
MAPK signaling pathway	21	21
mTOR signaling pathway	21	21
PI3K-Akt signaling pathway	21	21
Necroptosis	21	21
TGF-beta signaling pathway	21	21
Ubiquinone and other terpenoid-quinone biosynthesis	20	20
Methane metabolism	20	20
Wnt signaling pathway	20	20
Circadian rhythm - plant	20	20
Pentose phosphate pathway	19	19
Thiamine metabolism	19	19
Proteasome	19	19
Mismatch repair	19	19
Autophagy - other	19	19
Peroxisome	19	19
Ether lipid metabolism	18	18
Flavonoid biosynthesis	18	18
Lysosome	18	18
Apoptosis	18	18
beta-Alanine metabolism	17	17
RNA polymerase	17	17
Homologous recombination	17	17
FoxO signaling pathway	17	17
Fatty acid degradation	16	16
Terpenoid backbone biosynthesis	16	16
cGMP-PKG signaling pathway	16	16
Lysine degradation	15	15
Diterpenoid biosynthesis	15	15
Tropane, piperidine and pyridine alkaloid biosynthesis	15	15

Arginine and proline metabolism	14	14
Tryptophan metabolism	14	14
Folate biosynthesis	14	14
Hedgehog signaling pathway	14	14
Linoleic acid metabolism	13	13
alpha-Linolenic acid metabolism	13	13
Protein export	12	13
Citrate cycle (TCA cycle)	12	12
Porphyrin and chlorophyll metabolism	12	12
HIF-1 signaling pathway	12	12
Apelin signaling pathway	12	12
Focal adhesion	12	12
Carbon fixation pathways in prokaryotes	11	11
Sulfur metabolism	11	11
Metabolism of xenobiotics by cytochrome P450	11	11
Mitophagy - animal	11	11
Ferroptosis	11	11
Brassinosteroid biosynthesis	10	10
Calcium signaling pathway	10	10
Gap junction	10	10
Fatty acid biosynthesis	9	9
Steroid biosynthesis	9	9
Valine, leucine and isoleucine degradation	9	9
Hippo signaling pathway	9	9
Other glycan degradation	8	8
Propanoate metabolism	8	8
Zeatin biosynthesis	8	8
Basal transcription factors	8	8
Base excision repair	8	8
Aminobenzoate degradation	7	7
Styrene degradation	7	7
Pantothenate and CoA biosynthesis	7	7

Rap1 signaling pathway	7	7
Signaling pathways regulating pluripotency of stem cells	7	7
Cutin, suberine and wax biosynthesis	6	6
Other types of O-glycan biosynthesis	6	6
Biofilm formation - Escherichia coli	6	6
ErbB signaling pathway	6	6
VEGF signaling pathway	6	6
Adherens junction	6	6
TNF signaling pathway	6	6
Photosynthesis	5	5
Caffeine metabolism	5	5
Taurine and hypotaurine metabolism	5	5
Lipopolysaccharide biosynthesis	5	5
Sphingolipid metabolism	5	5
Nicotinate and nicotinamide metabolism	5	5
Retinol metabolism	5	5
Glucosinolate biosynthesis	5	5
Valine, leucine and isoleucine biosynthesis	4	4
Histidine metabolism	4	4
N-Glycan biosynthesis	4	4
Various types of N-glycan biosynthesis	4	4
Glycosaminoglycan degradation	4	4
Chloroalkane and chloroalkene degradation	4	4
Naphthalene degradation	4	4
Butanoate metabolism	4	4
Vitamin B6 metabolism	4	4
Lipoic acid metabolism	4	4
Monoterpenoid biosynthesis	4	4
Notch signaling pathway	4	4
Biofilm formation - Vibrio cholerae	4	4
Steroid hormone biosynthesis	3	3
Aflatoxin biosynthesis	3	3

Mannose type O-glycan biosynthesis	3	3
Streptomycin biosynthesis	3	3
Neomycin, kanamycin and gentamicin biosynthesis	3	3
Glycosylphosphatidylinositol (GPI)-anchor biosynthesis	3	3
Arachidonic acid metabolism	3	3
Biosynthesis of unsaturated fatty acids	3	3
Bacterial secretion system	3	3
Non-homologous end-joining	3	3
Sulfur relay system	3	3
Fatty acid elongation	2	2
Synthesis and degradation of ketone bodies	2	2
Geraniol degradation	2	2
Chlorocyclohexane and chlorobenzene degradation	2	2
Fluorobenzoate degradation	2	2
Phenazine biosynthesis	2	2
Selenocompound metabolism	2	2
D-Glutamine and D-glutamate metabolism	2	2
Polyketide sugar unit biosynthesis	2	2
Toluene degradation	2	2
Riboflavin metabolism	2	2
Biosynthesis of ansamycins	2	2
Biofilm formation - Pseudomonas aeruginosa	2	2
Jak-STAT signaling pathway	2	2
Photosynthesis - antenna proteins	1	1
Monobactam biosynthesis	1	1
Lysine biosynthesis	1	1
C5-Branched dibasic acid metabolism	1	1
Biotin metabolism	1	1
Atrazine degradation	1	1
Indole alkaloid biosynthesis	1	1
Two-component system	1	1
Neuroactive ligand-receptor interaction	1	1

Table S3. Plastid primers used to amplify the *Coffea* samples.

Gene	Primer sequences
matK	F: CGTACAGTACTTTTGTGTTTACGAG
	R: ACCCAGTCCATCTGGAAATCTTGGTTC
rbcL	F: ATGTCACCACAAACAGAGACTAAAGC
	R: GAAACGGTCTCTCCAACGCAT

Table S4. Taxa retrieved from NCBI and used in the phylogenetic analyses.

Genebank ID	Species
MK577911	*Coffea racemosa*
JX572420	*Coffea racemosa*
MK577910	*Coffea pseudozanguebariae*
MK577912	*Coffea stenophylla*
MK577913	*Coffea tetragona*
MK577907	*Coffea humblotiana*
KC758284	*Coffea arabica*
MN894552	*Coffea arabica*
AB973183	*Coffea arabica*
AB973185	*Coffea arabica*
AB973184	*Coffea arabica*
JN114797	*Coffea arabica*
MK875244	*Coffea arabica*
MK862266	*Coffea arabica*
NC008535	*Coffea arabica*
AB973187	*Coffea arabica*
AB973188	*Coffea arabica*
AB973186	*Coffea arabica*
MK577909	*Coffea macrocarpa*
GQ248573	*Coffea myrtifolia*
KC758293	*Coffea liberica*
KC758290	*Coffea liberica v. dewevrei*
KC758294	*Coffea canephora*
AB973189	*Coffea canephora*
KC758295	*Coffea canephora*
KC628267	*Psilanthus mannii*

Table S5. SSRs primers used to amplify the *Coffea* samples.

ID	Primer sequences
**CCRM02**	F: AATGGTGGCAGTCCTGAAAGATC
	R: AACATCAACTTTCCTGGTCTTC
**CCRM07**	F: TAAAGGATGGTATATGTGGCTGGAGTA
	R: CCACAGCCTCGGCATTTACTATATAT
**CCRM16**	F: TCCTATAGCAGAAACACAAAATGACACAG
	R: GGTTTTTGGGTTCTTTTTAGCATATACA
**CCRM17**	F: TAAGCGTTGGAATTCCTCACTCTATCT
	R: ACAGCTAAAGAAACAATGAACCAGT
**CC2P4**	F: TAGCAGCAAACACTCTTCGC
	R: GCTTCTGACGGACTTGAGGA
**CC5P3**	F: CTGTTACAGCCTCGTCCACT
	R: TCCCGCTACTTTCATGGGAT
**CC4P5**	F: CTAGTCTTGGCATGTTGGGG
	R: CTTTGCTGTGGAGGTAAGGC
**CC1P7**	F: GGGGCAAAACAAGAACCACT
	R: ACCTTATCCAAAACCCATGTGC
**CC3P6**	F: CTTGGGATTGCCTAGCCCTA
	R: TCTTGCCGTTTTAGCCGATT
**CC4P8**	F: TGAGAAGGGACAAAGAAAGAGG
	R: ACAGTACAACATATGAGGCCAC
**AJ308790**	F: TTTTCTGGGTTTTCTGTGTTCTC
	R: TAACTCTCCATTCCCGCATT
**AJ308779**	F: TCCCCCATCTTTTTCTTTCC
	R: GGGAGTGTTTTTGTGTTGCTT
**AJ308809**	F: AGCAAGTGGAGCAGAAGAAG
	R: CGGTGAATAAGTCGCAGTC
**SSR03**	F: GGACAAAACACCGCCCAAAATA
	R: AGCGAGACAGAGGAAGGGAATATT

Table S6. Summary of sequencing and mapping of reads from *Coffea arabica* and *C. racemosa* samples

Sample	Species	Raw Reads	Clean Reads	GC(%)	Q20	% mapping
**IR02**	*Coffea racemosa*	6395090	1753058	37.58	97.09	77.67%
**ZV01**	*Coffea racemosa*	8312096	2060734	37.11	96.15	75.59%
**HO04**	*Coffea racemosa*	7140830	2094006	38.61	96.42	83.51%
**MR01**	*Coffea racemosa*	5410602	1605185	36.47	96.11	78.62%
**MR02**	*Coffea racemosa*	5865342	1852596	37.78	96.05	82.58%
**MX05**	*Coffea racemosa*	7393166	2097160	36.32	95.31	84.04%
**MX03**	*Coffea racemosa*	4802168	1408845	37.23	95.13	84.82%
**SF05**	*Coffea racemosa*	8806602	2520889	37.10	96.91	76.07%
**MP05**	*Coffea racemosa*	5111332	1533854	36.71	96.36	83.34%
**MP04**	*Coffea racemosa*	8802762	2406989	37.67	95.80	82.94%
**MR03**	*Coffea racemosa*	4277686	1348832	37.81	95.16	79.69%
**MP08**	*Coffea racemosa*	4006454	1529142	37.53	96.09	81.00%
**HO03**	*Coffea racemosa*	4604378	1332536	37.61	96.71	77.52%
**IR01**	*Coffea racemosa*	5766290	1554729	37.05	96.49	74.60%
**Ca-A650-P3**	*Coffea arabica*	5843528	1545682	38.28	94.54	80.53%
**Ca-A650-P6**	*Coffea arabica*	6184428	1855793	38.28	96.02	81.05%
**Ca-A650-P7**	*Coffea arabica*	7465534	2325304	38.41	96.33	86.94%
**Ca-A650-P8**	*Coffea arabica*	6732436	2035553	38.27	96.93	86.26%
**Ca-A650-P11**	*Coffea arabica*	4990690	1516133	38.15	94.91	77.48%
**Ca-A825-P16**	*Coffea arabica*	4524368	1424617	38.06	95.94	83.28%
**Ca-A825-P18**	*Coffea arabica*	5713418	2837142	45.73	96.14	28.89%
**Ca-A825-P22**	*Coffea arabica*	7341088	2468541	38.59	97.25	82.10%
**Ca-A825-P23**	*Coffea arabica*	5385822	1576465	38.72	94.75	78.26%
**Ca-A825-P16**	*Coffea arabica*	4524368	1424617	38.06	95.94	83.28%
**Ca-P935-P33**	*Coffea arabica*	4686380	1457994	38.34	94.98	82.93%
**Ca-P935-P37**	*Coffea arabica*	6320542	1694251	38.74	96.59	76.10%
**Ca-P935-P38**	*Coffea arabica*	8754090	2454975	37.79	97.15	85.33%
**Ca-P935-P41**	*Coffea arabica*	5914830	1555252	38.11	96.31	86.50%
**Ca-P935-P43**	*Coffea arabica*	4168100	1269412	37.37	96.15	85.46%
